# Chitosan Nanoparticles Inactivate Alfalfa Mosaic Virus Replication and Boost Innate Immunity in *Nicotiana glutinosa* Plants

**DOI:** 10.3390/plants10122701

**Published:** 2021-12-08

**Authors:** Ahmed Abdelkhalek, Sameer H. Qari, Mohamed Abd Al-Raheem Abu-Saied, Abdallah Mohamed Khalil, Hosny A. Younes, Yasser Nehela, Said I. Behiry

**Affiliations:** 1 Plant Protection and Biomolecular Diagnosis Department, ALCRI, City of Scientific Research and Technological Applications, New Borg El-Arab City 21934, Alexandria, Egypt; 2 Biology Department, Al-Jumum University College, Umm Al-Qura University, Mecca 25376, Saudi Arabia; shqari@uqu.edu.sa; 3 Polymeric Materials Research Department, Advanced Technology and New Materials Research Institute, City of Scientific Research and Technological Applications (SRTA-City), New Borg El-Arab City 21934, Alexandria, Egypt; Maabusaied@srtacity.sci.eg; 4 Plant Botany Department, Faculty of Science, Omar Al-Mukhtar University, Al Bayda 00218-84, Libya; abdallak805@gmail.com; 5 Agricultural Botany Department, Faculty of Agriculture (Saba Basha), Alexandria University, Alexandria 21531, Egypt; hosnyyounes@yahoo.com; 6 Department of Agricultural Botany, Faculty of Agriculture, Tanta University, Tanta 31511, Egypt; yasser.nehela@ufl.edu; 7 Citrus Research and Education Center, Department of Plant Pathology, University of Florida, 700 Experiment Station Rd., Lake Alfred, FL 33850, USA

**Keywords:** chitosan nanoparticles, alfalfa mosaic virus, antiviral activity, gene expression

## Abstract

Plant viral infection is one of the most severe issues in food security globally, resulting in considerable crop production losses. Chitosan is a well-known biocontrol agent against a variety of plant infections. However, research on combatting viral infections is still in its early stages. The current study investigated the antiviral activities (protective, curative, and inactivation) of the prepared chitosan/dextran nanoparticles (CDNPs, 100 µg mL^−1^) on *Nicotiana glutinosa* plants. Scanning electron microscope (SEM) and dynamic light scattering analysis revealed that the synthesized CDNPs had a uniform, regular sphere shapes ranging from 20 to 160 nm in diameter, with an average diameter of 91.68 nm. The inactivation treatment was the most effective treatment, which resulted in a 100% reduction in the alfalfa mosaic virus (AMV, Acc# OK413670) accumulation level. On the other hand, the foliar application of CDNPs decreased disease severity and significantly reduced viral accumulation levels by 70.43% and 61.65% in protective and curative treatments, respectively, under greenhouse conditions. Additionally, the induction of systemic acquired resistance, increasing total carbohydrates and total phenolic contents, as well as triggering the transcriptional levels of peroxidase, pathogen-related protein-1, and phenylalanine ammonia-lyase were observed. In light of the results, we propose that the potential application of CDNPs could be an eco-friendly approach to enhance yield and a more effective therapeutic elicitor for disease management in plants upon induction of defense systems.

## 1. Introduction

Plant viruses are one of the main plant pathogens that pose a severe threat to sustainable agriculture and food security throughout the world [1]. Among them, the Alfalfa mosaic virus (AMV) is one of the most significant and widely spread viruses in Egypt, causing severe crop losses [2,3]. AMV can infect more than 430 plant species belonging to 51 families, causing mild to severe leaf symptoms, dark green mottle counting, brilliant yellow mottle, necrotic or chlorotic lesions, leaf deformation, vein necrosis, and ring spots, among others [2,4]. In nature, AMV is transmitted in a non-persistent manner by sap inoculation and a variety of 25 aphid species, as well as by seeds and pollen in some plant species [5]. Under greenhouse conditions, AMV-like symptoms were clearly observed after 14 to 21 days of viral inoculation depending on the type of host plant and cultivar [3,6,7,8]. Due to limited control methods, new viral disease management strategies have been proposed in order to achieve better and more sustainable viral disease control. Such ways depend on promoting natural plant defense, e.g., systemically acquired resistance (SAR) [9,10]. The early recognition of pathogens is critical for plants' natural defense mechanism against pathogenesis. Commonly, the stimulation of a natural defense response involves increased expression of defense-related genes and enzymes as well as increased phenolic compound accumulation [11]. Plants treated with various biotic elicitor molecules have been demonstrated to elicit this innate immune response the same way that infections do [12,13].

Chitosan, a natural polymer, has been found to be an efficient biotic elicitor in plants causing systemic resistance development [14]. Chitosan is a well-known biocontrol agent and abundant dual-effect natural polymer due to its non-toxic, biodegradable, and biocompatible characteristics [15]. Chitosan application could prevent the microbial pathogens from growing and sporulating by disrupting the membranes of their cells and inhibiting various biochemical processes during the plant–pathogen interaction, which causes distinct defensive responses in host plants. Because of the challenges and unique needs of growing viruses, chitosan's antiviral activity (and derivatives) has received little attention. However, some information about chitosan's antiviral action may be found in the literature, and several studies have shown that chitosan protects plants from viral infection [16,17,18]. Chitosan, for example, has been shown to successfully induce viral resistance in plants such as tomatoes, cucumbers, potatoes, sunflowers, and tobacco [17,18]. It was reported that the chitosan biopolymer's unique features could be further strengthened by employing them as nanoparticles. In this form, it can impart varied biological activities with altered physicochemical qualities such as size, cationic nature, and surface area [19]. In this context, nanomaterials have emerged as a potential technical innovation that has the ability to alter the agriculture sector by enhancing crop productivity, promoting plant-systemic resistance, and combating phytopathogens.

Several studies have been published on the up-regulation of defense-related enzymes/genes in several crop plants by chitosan nanoparticles [19,20,21,22]. Compared to chitosan, the foliar application of finger millet plants with chitosan nanoparticles (0.1%, *w/v*) significantly enhanced growth, yield, mineral content, and several defense enzymes such as peroxidase, chitinase, and polyphenol oxidase [23]. Furthermore, the biostimulant activity of chitosan nanoparticles in a variety of agricultural crop plants was reported [21,22,23,24] However, research to combat viral infections is still beginning [25,26]. Consequently, the current study aimed to synthesize and characterize chitosan/dextran nanoparticles (CDNPs) using dynamic light scattering (DLS), scanning electron microscope (SEM), and Fourier transform-infrared (FTIR) spectroscopy. In addition, a range of immune-related responses to differing CDNP treatments (protective, curative, and inactivating) was evaluated. To this end, we studied the effects of CDNPs on inducing SAR and transcriptional levels of defense-related genes (peroxidase (*POD*), pathogen-related protein-1 (*PR-1*), and phenylalanine ammonia-lyase (*PAL*)), as well as total carbohydrates and total phenolic content.

## 2. Materials and Methods

### 2.1. Preparation of Chitosan/Dextran Nanoparticles

Chitosan (molecular weight: 100,000–300,000), dextran sulfate, glacial acetic acid extra pure (99.5–100%, M.W = 60.05), and sodium hydroxide (NaOH) assay (97%, Fisher Chemical, Chicago, IL, USA) were used to prepare chitosan dextran nanoparticles (CDNPs) via the ionic-gelation method [27]. Briefly, 0.2% *w/v* chitosan solution in 1% *v/v* glacial acetic acid was prepared and adjusted to the pH value of 5 by adding, drop by drop, 1N NaOH. On the other hand, 0.2% *w/v* dextran solution in distilled water was prepared. Dextran solution was added slowly drop by drop into the chitosan solution at a 1:2 ratio under magnetic stirring at 700 rpm for 30 min to form CDNPs. After that, the obtained CDNPs were further characterized.

### 2.2. Characterization of Chitosan/Dextran Nanoparticles

A scanning electron microscope (SEM) was used to examine the morphology of the nanoparticles. The nanoparticle sample was placed on a self-adhesive carbon disc and placed on a 25 mm aluminum stub with a sputter coater. The stub was coated with 25 nm gold and then imaged on an FEI Quanta 200 FEG (SEM) at 5kV. Finally, secondary electron detection was used to accelerate voltages [28]. A Fourier Transform Infra-Red (FTIR) spectroscopy instrument (Shimadzu FTIR-8400 S, Japan) was used to investigate different functional groups of the prepared CDNPs. The samples were ground gently with 300 mg of micronized potassium bromide (KBr) powder and compressed into discs at a force equal to 10 kN for 2 min. A 256-scan interferogram was collected for each spectrum at room temperature to investigate the success of the synthesis process [29].

### 2.3. Viral Isolation and Molecular Characterization

Samples of potatoes (*Solanum tuberosum* L.) exhibited characteristics of AMV-like symptoms, including brilliant mottling and yellow blotching, and were obtained from Egypt's farms. All samples were analyzed for viral presence using Double Antibody Sandwich-Enzyme-Linked Immunosorbent Assay (DAS-ELISA) with specific antiserum (DSMZ, AS-0779) [30,31]. The purified single local lesion developed on *Chenopodium amaranticolor* leaves, as a local lesion host for AMV, was used as a viral source to inoculate *Nicotiana glutinosa* plants in an insect-proof greenhouse. According to the manufacturer's instructions, the QIAamp Viral RNA Kit was used to extract viral RNA (QIAGEN, Hilden, Germany). As previously described [32,33], first-strand cDNA was generated and subjected to PCR to amplify the *AMV-CP* gene before sending it to sequence at Macrogen company (Seoul, Korea). The nucleotide sequence was deposited in GenBank to obtain the accession number.

### 2.4. Greenhouse Antiviral Activity Assays and Experimental Design

The uniform *N. glutinosa* seeds were surface sterilized and cultivated in plastic pots (25 cm in diameter) filled with sterilized soil. At the 5–6th leaf stage, *N. glutinosa* seedlings were transported to new pots; each pot contained three plants, and after one week of transplantation, plants of similar sizes were subjected to antiviral assays. The purified inoculum concentration of AMV was diluted to 20 µg mL^−1^ with 0.01 M phosphate buffer before use, while CDNPs (0.01%, *w/v*) were dispersed in sterile distilled water. As previously described [34], two true upper leaves of each plant were dusted with carborundum (600 mesh) and mechanically inoculated with the virus-inoculum (100 µL/leaf). Five treatments—control (mock-inoculated control), infected (AMV-inoculated control), protective, curative, and inactivating—were performed on *N. glutinosa* plants. The *N. glutinosa* plants treated with CDNPs (100 µg mL^−1^) 24 h before AMV inoculation served as a protective treatment. In comparison, plants treated with CDNPs (100 µg mL^−1^), 24 h after AMV mechanical inoculation, were used as curative treatment. For inactivating treatment, an equal volume of purified AMV (20 µg mL^−1^) and CDNPs (100 µg mL^−1^) was mixed and incubated for 1 h before mixture inoculation (200 µL/leaf), and CDNPs foliar application. The whole plant shoots were foliar sprayed until run-off, and the leaves appeared to be coated with the solution using a handheld pressure sprayer. The *N. glutinosa* plants inoculated with AMV alone were used as an infected treatment. As a negative control, mock-treated plants were inoculated with viral inoculation buffer and foliar sprayed with sterile distilled water. All plants were kept under greenhouse conditions (28 °C/16 °C, 16 h/8 h light/dark cycle, and relative humidity of 70%), and symptom development was recorded regularly for 20 days. For further analysis, three independent biological replicates of *N. glutinosa* leaves of all treatments were collected at 3, 6, 10, 15, and 20 days post-inoculation (dpi). The DAS-ELISA test was performed on all the treatments to determine AMV systemic accumulation level as previously described [30].

### 2.5. Estimation of Total Phenolic Content

Total phenolic content (TPC) was estimated as described by Jindal and Singh [35]. Five hundred milligrams of dried plant tissues were extracted with absolute ethanol, centrifuged, and the supernatant was mixed with a mixture of Folin–Ciocalteu reagent and sodium carbonate 20% (1:1 *v/v*). The combination was completed with 10 mL of deionized water and kept in the dark for 30 min. A double beam spectrophotometer (OPTIMA, Japan) determined the absorbance values at 650 nm. Different concentrations of pyrogallol were used to prepare the standard curve, and TPC values were determined as mg g^−1^ DM in the plant shoot.

### 2.6. Determination of Total of Soluble Carbohydrates

The phenol sulfuric acid method was used to estimate the total soluble carbohydrates, according to Dubois et al. [36]. *N. glutinosa* dried plants were ground to a fine powder. In the test tube, 0.1 g of plant powder was added to a 5 mL borate buffer containing 28.63 g boric acid, 29.8 g potassium chloride, and 3.5 g sodium hydroxide/L, pH 8.0. The tubes were kept for 24 h at 4 °C, then centrifuged for 15 min at 3000 rpm. The collected supernatants were combined with 6 mL of a mixture of phenol 5% and sulfuric acid (1:5) and placed in a water bath at 30 °C for 20 min. The resultant color was measured at 490 nm. The total soluble carbohydrate content values were determined as mg g^−1^ DM using a calibration curve of glucose sugar.

### 2.7. Transcriptional Levels of Defense-Related Genes

By using the guanidium isothiocyanate extraction method [37,38], 100 mg of *N. glutinosa* leaves collected at 3, 6, 10, 15, and 20 dpi for all treatments was utilized as starting material for total RNA extraction. After checking the concentration, purity, and integrity of the extracted RNA on Nano SPECTROstar and agarose gel electrophoresis, respectively, 1 μg of DNase-treated total RNA from each sample was used as a template for cDNA synthesis as previously described [39]. The reverse transcription reaction was incubated at 42 °C for 1 h, then inactivated at 80 °C for 7 min and stored at −20 °C until used. For detecting the expression profiles of the three *N. glutinosa* genes (*PAL*, *PR-1*, and *POD*), the synthesized cDNA was subjected to qRT-PCR amplification using a specific primer (Table 1). A real-time cycler (Corbett Rotor-Gene Q, Qiagen, Manchester, UK) was used to amplify all genes transcripts with program features as described previously [40]. The *β-actin* reference gene (Table 1) was applied to normalize the transcript expression of each gene. The qRT-PCR amplification program consisted of an initial denaturation at 95 °C for 10 min, followed by 45 cycles programmed as 95 °C for 20 s, 60 °C for 30 s, and 72 °C for 30 s. Reactions of each sample were performed with three technical replicates. The relative expression levels were quantified according to Livak and Schmittgen [41].

### 2.8. Statistical Analyses

Data of ELISA, total carbohydrates, and total phenolics were statistically analyzed according to the analysis of variance technique (ANOVA), followed by post hoc pairwise comparisons between them using the Tukey–Kramer honestly significant difference test (Tukey HSD, *p* ≤ 0.05). Relative expression values of different defense-related genes (*POD*, *PR-1*, and *PAL*) were analyzed using full factorial ANOVA based on a split-plot design with five treatments (mock-inoculated control, AMV-inoculated control, protective, curative, and inactivating) in the main plots and six-time points (0, 3, 6, 10, 15, 20 days post-inoculation) in subplots. Compared to mock-inoculated control treatment, the relative transcriptional values higher than 1 were classified as elevations in gene expression (up-regulation/induction). In contrast, values lower than 1 were classified as declines in expression levels (down-regulation/suppression). In addition, based on the assumptions of linearity, simple linear regression (SLR) analysis was performed to model the relationship between time post-inoculation (as an independent variable) and relative expression levels of different defense-related genes including *POD*, *PR-1*, and *PAL* (as dependent variables). The fitted SLR line is expressed by the equation determined by the F test (*p* ≤ 0.05). Both the coefficient of determination (R^2^) and the adjusted coefficient of determination (R^2^_adj_) were also obtained. Additionally, because of the experiential nonlinear phenomena between time post-inoculation and relative expression levels of different defense-related genes (*POD*, *PR-1*, and *PAL*), and to better understand the curvilinear relationship between them, data were fitted with a second-degree polynomial regression model (quadratic model). Quadratic equation, R^2^, R^2^_adj_, and *p*-value based on the F test (*p* ≤ 0.05) were also obtained. Both SLR and polynomial regression were carried out using JMP 15 Software from SAS (https://www.jmp.com/en_us/home.html accessed on 3 December 2021). 

## 3. Results and Discussion

### 3.1. Structural, Compositional Characterization, and Particle Size of the Synthesized CDNPs

**Figure 1:**
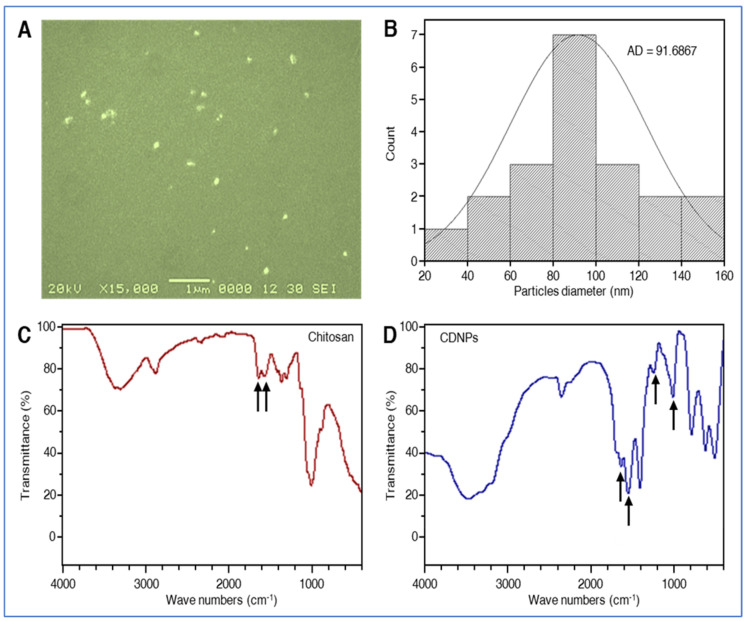
Structural and compositional characterization of chitosan/dextran nanoparticles (CDNPs). (**A**) SEM image of the prepared CDNPs, (**B**) particle size distribution of the synthesized CDNPs using dynamic light scattering technique. (Bar = 1 µm at ×15,000), (**C**,**D**) FTIR spectra of pure chitosan and CDNPs, respectively.

Scanning electron microscopy (SEM) is a widely used method for determining the surface morphology of nanoparticles [26]. In the present study, the SEM analysis showed that the CDNPs have uniform, regular, and spherical shapes with a little aggregation between them (Figure 1A). Dynamic light scattering (DLS) is a popular method for determining particle size distribution in aqueous/colloidal solutions [42]. The DLS analysis revealed that the diameter range of the prepared CDNPs was between 20 and 160 nm, with an average diameter of 91.68 ± 4.87 nm (Figure 1B).

### 3.2. Infrared Spectrophotometry

The FTIR spectra of pure chitosan (Figure 1C) exhibited characteristic broad band signals at 3346–3286 cm^−1^ referred to N-H and O-H groups, respectively [43]. A peak of amide I band appears at 1641 cm^−1^, and a strong protonated amino peak is at 1575 cm^−1^ [44]. Around 1370 cm^−1^ shows a peak corresponding to C-N stretching, and C–O–C asymmetric stretching was found around 1143 cm^−1^ [45]. In dextran, sulfyl peaks presented at 1026 cm^−1^ and 1261 cm^−1^ due to symmetric and asymmetric stretching vibrations of (SOO-), and the band around 820 cm^−1^ correspondents to S–O–S vibrations [46]. In chitosan/dextran sulfate nanoparticles, the FTIR also shows a peak of amide bond at 1642 cm^−1^, and the strong protonated amino peak shifted from 1575 to 1559 cm^−1^ (Figure 1D). There was a peak shift in the sulfate stretching vibration spectrum from 1261 to 1256 cm^−1^. Changes in the amine group of chitosan and the sulfate group of dextran are caused by inotropic interaction between cationic and anionic molecules, respectively [45]. The FTIR spectra clarified the successful cross-linking between chitosan and dextran to form CDNPs.

### 3.3. Viral Isolation and Molecular Characterization

**Figure 2:**
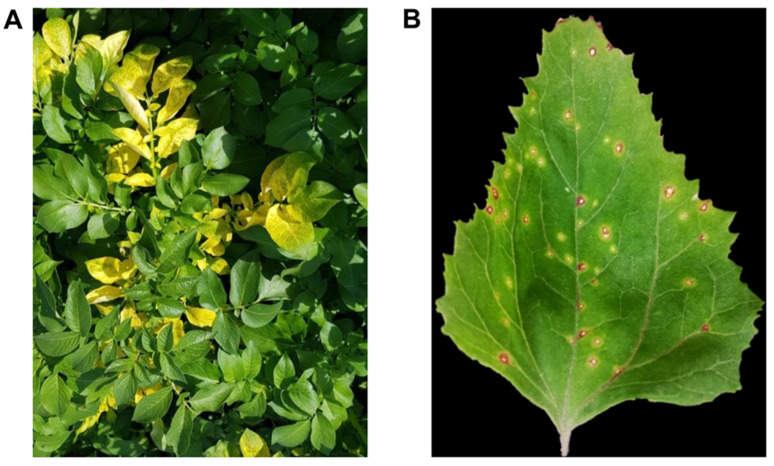
(**A**) Calico symptoms on naturally AMV-infected potato (*Solanum tuberosum* L.) plants; (**B**) local lesions developed on *Ch. amaranticolor* leaves in response to AMV after mechanical inoculation at 5 dpi.

The yellow blotching and bright mottling symptoms on the leaves were the common AMV symptoms in the field-collected potato (*Solanum tuberosum* L.) samples (Figure 2A). Most potato cultivars are susceptible to AMV infection, resulting in a variety of symptoms ranging from bright yellow blotching and mottling to clear visibility of calico symptoms [2,3,47]. At 4–5 dpi, single local lesions developed on *Chenopodium amaranticolor* leaves [48] and were used as a source of pure AMV isolate for purification and antiviral assay studies (Figure 2B). The sequence information of the viral coat protein (CP) gene is a very important criterion for plant virus identification and taxonomy. Bromoviridae have a lot of variation in their CP; in addition, it is the most useful molecule for their phylogeny [49].

**Figure 3:**
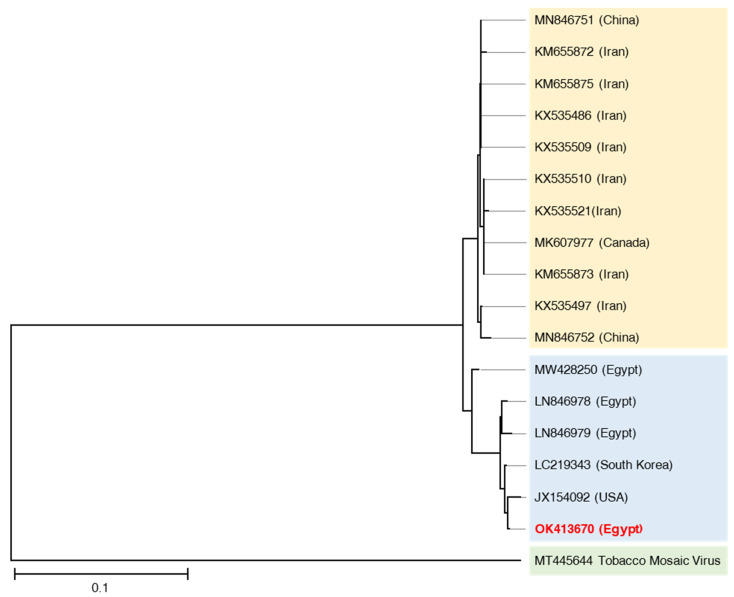
Phylogenetic tree based on the nucleotide sequence of the coat protein (CP) gene of the Egyptian alfalfa mosaic virus isolate OK413670, and other CP genes of AMV isolates retrieved from GenBank. The phylogeny was created using the UPGMA statistical approach and tested using the bootstrap method with 2000 replications.

In the present study, RT-PCR was performed on the extracted total RNA from infected plant tissues with specific *AMV-CP* gene primers. Analysis of PCR products in agarose gel electrophoresis revealed that the amplification of a specific band was approximately 350 bp, which was in agreement with the size of the fragment expected from the sequence data reported previously [31]. After PCR product purification and sequencing, the annotated sequence was deposited in the GenBank database under isolate ASHA1 and accession number OK413670. According to NCBI-BLAST alignment, our Egyptian isolate (OK413670) shared the highest similarity of 99% with the USA isolate (JX154092) and South Korea isolate (LC219343) while sharing a similarity of 98% with other isolates from Egypt (LN846978 and LN846979). Moreover, isolate MW428250 showed a lower similarity of 97% with our isolate reported in this study. This result indicates that AMV isolates in Egypt have a lot of genetic variabilities. Moreover, the phylogenetic tree (Figure 3) analysis showed that the aligned AMV isolates were clustered into two subgroups, which could be related to their geographic origin [50].

### 3.4. Antiviral Activity of Chitosan/Dextran Nanoparticles (CDNPs)

#### 3.4.1. Effect of CDNPs on Disease Severity and AMV Systemic Accumulation Level

**Figure 4:**
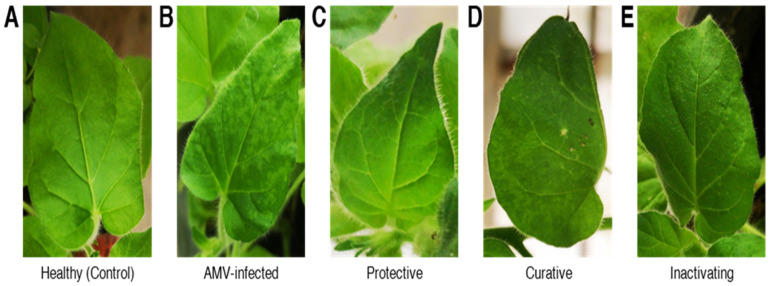
Effect of chitosan/dextran nanoparticles (CDNPs) on the disease symptoms development on *N. glutinosa* leaves infected with AMV at 20 days post-inoculation. (**A**) Mock-inoculated control plants, (**B**) AMV-inoculated control plants, (**C**) plants treated with CDNPs (100 µg mL^−1^) 24 h before inoculation of AMV (protective treatment), (**D**) plants treated with CDNPs (100 µg mL^−1^) 24 h after inoculation of AMV (curative treatment), (**E**) plant treated with a mixture of CDNPs with the same amount of purified TMV and incubated for 1 h (inactivity treatment).

Under greenhouse conditions, the antiviral activity (protection, curation, and inactivation) of the prepared CDNPs against AMV on *N. glutinosa* was evaluated. The application of CDNPs (100 µg mL^−1^) significantly reduced the disease severity and decreased the AMV accumulation levels of the CDNPs-treated plants when compared to AMV-inoculated control treatment plants. At 20 dpi, and compared with mock-inoculated control (Figure 4A), the AMV-inoculated *N. glutinosa* control plants showed severe mosaic symptoms (Figure 4B). On the other hand, a delay in the appearance of the symptoms for four days was observed in CDNPs-treated plants 24 h before viral inoculation (protective treatment; Figure 4C). Additionally, plants treated with CDNPs 24 h after viral inoculation (curative treatment) showed mild mosaic symptoms compared to AMV-inoculated control plants (Figure 4D). No symptoms were observed on either mock-inoculated control or inactivation treatment plants (Figure 4A,E, respectively). It was observed that foliar treatment of tomato and tobacco plants with chitosan (0.1 %, *w/v*) 24 h before CMV inoculation was associated with no deleterious symptom development [51]. On the other hand, spraying coffee seedlings with chitosan nanoparticles in the range of 420 to 970 nm for three times at a concentration of 10 ppm improved chlorophyll and carotenoid contents, uptake of nitrogen and magnesium, as well as the growth of coffee in the pots under greenhouse conditions [52]. Compared to bulk chitosan, the foliar application of chitosan nanoparticles (0.1 %, *w/v*) significantly enhanced finger millet growth, yield, and mineral content [23]. Foliar-sprayed nanoparticles mainly enter the plant leaves through stomata or epidermal adsorption and are transported to various plant parts via the vascular system and symplastic pathways [53]. Consequently, the inactivation activity of the prepared CDNPs reflects the direct activity against AMV particles through binding to RNA, resulting in replication inhibition [54,55]. On the other hand, CDNPs may be acting as elicitor molecules, activating plant immunity, and triggering the hypersensitivity response through increased antioxidant and defensive enzyme activity [56].

**Figure 5:**
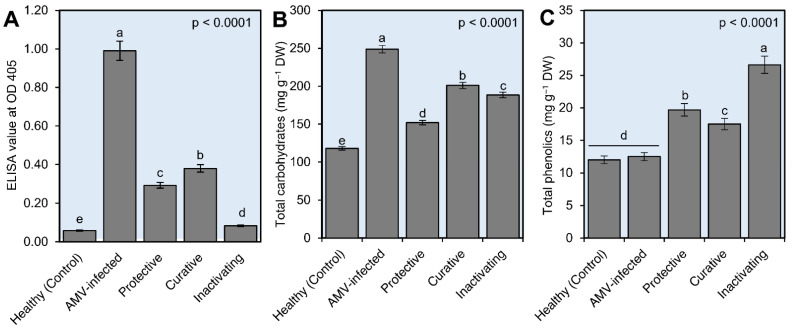
(**A**) A histogram showing the accumulation level of AMV in AMV-infected *N. glutinosa* plants at 22 dpi of different treatments. (**B**) Effect of chitosan/dextran nanoparticles (CDNPs) on total carbohydrates and total phenolics (**C**) of AMV-infected *N. glutinosa* plants at 22 dpi of different treatments. Data presented are means ± standard deviation (mean ± SD) of three biological replicates. Different letters indicate statistically significant differences among treatments according to Tukey’s honestly significant difference test (*p* < 0.05).

The ELISA test was performed on all the treatments, and the results were consistent with the appearance of symptoms in terms of the highest concentration and the lowest and free of the virus. The highest viral concentration level was observed in AMV-inoculated control plants (non-CDNPs treated) with an ELISA value of 0.991, while the mock-inoculated control plants showed a 0.0585 ELISA value (Figure 5A). The CDNPs-treated plants exhibited ELISA values of 0.293, 0.380, and 0.083 for curative, protective, and inactivating treatments, respectively (Figure 5A). The ELISA reactions revealed that the inactivating treatment was the most effective CDNPs treatment, followed by protective and curative treatments. The considerable reduction in AMV accumulation level in *N. glutinosa* leaves by 100%, 70.43%, and 61.65% in inactivating, protective, and curative treatments, respectively, reflected the anti-AMV activity of the prepared CDNPs nanoparticles. In this context, foliar application and treatment of tomato plants with ZnO NPs and Ag NPs reduced viral accumulation levels inside plant tissues and decreased disease severity [26,57]. In agreement with our results, chitosan nanoparticles might attach to virus particles, inhibit nucleic acid replication inside infected cells, as well as boost plant immunity and antioxidant defense systems [51,58]. Electron microscope examination revealed that the TMV particles were directly affected by nanoparticles, resulting in decreased viral particles, and the majority of them twisted together and tied into a bundle, leading to virus structure rupture [55,59]. In agreement with our results, the DAS-ELISA showed the efficacy of chitosan to control CMV, PVX, and TMV infections [51,58,60]. The authors suggested that chitosan increased plant resistance through enhancing ribonuclease activity and callose deposition as well as increasing the activity of hydrolases (proteases and RNases). Consequently, the data obtained support the beneficial effects of CDNPs as plant viral inhibitors as well as plant resistance inducers.

#### 3.4.2. Determination of Total Soluble Carbohydrates and Total Phenolic Contents

The plant accumulates soluble carbohydrates throughout development and maturation, which are involved in most basic physiological processes and have important roles in seed germination and seed desiccation tolerance [61]. Carbohydrate changes directly correlate with physiological processes such as photosynthesis, transpiration, and respiration [62]. Stress conditions alter carbohydrate accumulation and distribution in plants [63]. The present study clearly showed that CDNPs treatments generally induced a significant increase in total soluble carbohydrates. The highest total soluble carbohydrate (747 mg g^−1^ DM) was reported in the AMV-inoculated control plants, followed by curative, inactivating, and protective treatments with 603, 565, and 456 mg g^−1^ DM, respectively. Moreover, the mock-inoculated control plants exhibited 354 mg g^−1^ DM (Figure 5B). Similar findings were reported by Hoekstra et al. [64], who observed that the soluble carbohydrates accumulated in accordance with plant abiotic stress. Although some plant viruses upon infection have no effect on carbohydrate synthesis or its translocation in the leaf tissues, other viruses may modulate it [65]. The accumulation of carbohydrates (starches) has indeed been recognized to anticipate the appearance of virus symptoms in plants infected with ZYMV [65]. Arias et al. [66] reported similar results in sunflower plants infected with the sunflower chlorotic mottle virus. In addition, fructose and glucose sugars were greatly increased in leaves infected by the beet yellows virus [67]. In our study, we hypothesized that the CDNPs could alter the effects of viral infection on *N. glutinosa* by decreasing the total soluble carbohydrate content accumulation.

For total phenolic contents (TPC) estimation, the highest value (79.9 mg g^−1^) was reported in the inactivating treatment, while the lowest (36 mg g^−1^ DM) was reported in the mock-inoculated control treatment. Additionally, both protective and curative treatments showed increases in TPC with 59.1 and 52.5 mg g^−1^ DM, respectively, compared with AMV-inoculated control plants (37.7 mg g^−1^ DM; Figure 5C). In addition, data analysis revealed an increase in TPC following plant infection, which aligned with Khalid et al. [68], who found that phenolics were boosted in diverse plants following pathogen infection. Increases in plant metabolites such as phenols have been demonstrated to play a major part in the plethora of host–pathogen interactions, disease progression, and infected plant defensive system responses [69,70,71,72]. Based on the findings, we believe that the synthesized CDNPs have substantial antiviral efficacy that could be used in biocontrol agents as elicitor molecules to trigger SAR and plant viral disease management.

#### 3.4.3. Transcriptional Levels of the Defense-Related Genes

The transcription levels of three *N. glutinosa* defense-related genes, including peroxidase (*POD*), pathogenesis-related protein 1 (*PR-1*), and phenylalanine ammonia-lyase (*PAL*), were investigated at different time intervals of 3, 6, 10, 15, and 20 dpi.

##### Peroxidase (POD)

**Figure 6:**
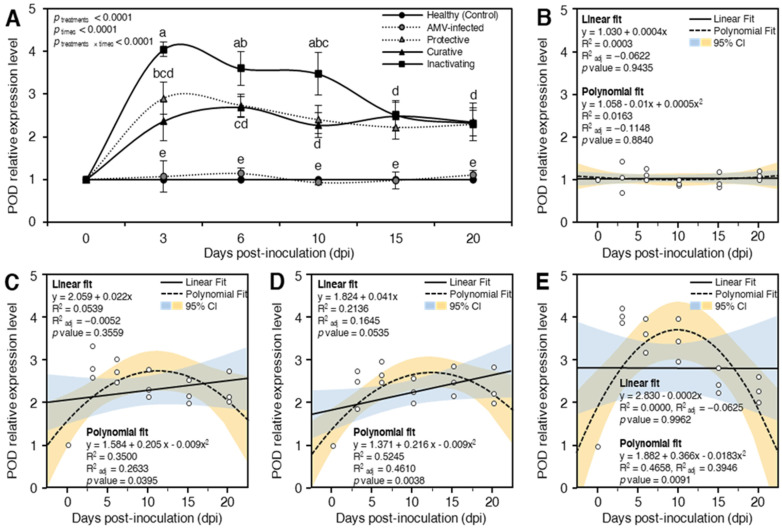
Effect of chitosan/dextran nanoparticles (CDNPs) on the relative expression level of peroxidase (*POD*) in AMV-infected *N. glutinosa* plants at 3, 6, 10, 15, and 20 dpi. (**A**) *POD* relative expression levels of different treatments at 3, 6, 10, 15, and 20 dpi. Data presented are means ± standard deviation (mean ± SD) of three biological replicates. Different letters indicate statistically significant differences among treatments according to Tukey’s honestly significant difference test (*p*_time × treatment_ < 0.05). (**B**–**E**) Simple linear regression (SLR) and quadratic polynomial regression analysis between *POD* relative expression and time post-inoculation of AMV-infected, protective-treated, curative-treated, and inactivating-treated plants, respectively. Open small circles present the row data (*n* = 3), solid lines present the SLR line, and polynomial regression models are presented as dashed-line. The 95% confidence intervals for the estimated regression are blue- and yellow-shaded. Regression equations, R^2^, R^2^_adj_, and *p*-value based on the F test (*p* < 0.05) were also obtained and presented within the graph.

It is well established that antioxidant enzymes play critical roles in preventing the serious consequences of reactive oxygen species (ROS) generated by viral infections, including plant cell damage [73,74]. For example, *POD* enzymes can remove H_2_O_2_, reduce free radicals, and protect the cytoplasmic membrane [75]. Compared to mock-inoculated control plants in the current study, the transcripts of *POD* were significantly induced after challenging *N. glutinosa* plants with CDNPs at various time intervals in different treatments (Figure 6A). Generally, the inactivating treatment had the highest *POD* expression levels throughout the experiment. At 3 dpi, the inactivating treatment showed the highest relative expression level (4.04-fold) followed by protective treatment (2.89-fold) and curative treatment with a relative expression level of 2.37-fold higher than mock-inoculated control (Figure 6A). At 6 dpi, the most outstanding relative transcriptional level (3.60-fold) was reported in the inactivating treatment, while protective and curative treatments showed 2.72- and 2.69-fold greater changes, respectively, than mock-inoculated control. At all studied times, 3, 6, 10, 15, and 20 dpi, no significant differences were reported between the AMV-inoculated control plants and mock-inoculated control plants (Figure 6A). It was stated that the induction and increasing activity of *POD* were associated with the increasing chlorophyll content and enhanced plant resistance against pathogens, including Mungbean yellow mosaic virus and TMV [76,77]. Moreover, POD was reported as a key player in plant defense responses against viral infection such as Pepper yellow mosaic virus in chili pepper (*Capsicum baccatum* var. *pendulum*) [78], cowpea chlorotic mottle virus in soybean (*Glycine max*) [79], and Tobacco mosaic virus (TMV) in tobacco plants [80]. In addition, foliar application of chitosan nanoparticles significantly elevated plant defense enzymes [23]. It was reported that chitosan nanoparticles can act as an immunological modulator in tea and finger millet plants by inducing antioxidant/defense enzyme activity [19,81]. At 10 dpi, the relative expression levels of protective, curative, and inactivating treatments were 2.40-, 2.26-, and 3.48-fold higher than mock-inoculated control, where at 15 dpi, they recorded 2.23-, 2.49-, and 2.52-fold changes, respectively, greater than mock-inoculated control (Figure 6A). At 20 dpi, the three treatments protective, curative, and inactivating exhibited transcriptional levels of 2.29-, 2.35-, and 2.32-fold change, respectively, with no significant change between them (Figure 6A).

Moreover, to better understand the relationship between *POD* relative expression and time post-inoculation, data were fitted using a simple linear regression (SLR) model. Although the SLR showed no correlation between *POD* relative expression of AMV-infected control treatment and time post inoculation (R^2^ = 0.0003 and *p* = 0.9435; Figure 6B), it showed a slight correlation between them in protective treatment (R^2^ = 0.0539 and *p* = 0.3559; Figure 6C). Additionally, the correlation between *POD* expression and time post inoculation was strengthened when *N. glutinosa* plants were treated with curative treatment (R^2^ = 0.2136 and *p* = 0.0535; Figure 6D), but not in the inactivating treatment (R^2^ = 0.0000 and *p* = 0.9962; Figure 6E). In the current study, all CDNPs treatments substantially increased the expression of *POD*; however, only protective and curative, but not inactivating, treatments showed a positive relationship with time post-inoculation with its highest peak at 3 dpi. Furthermore, due to the nonlinear phenomena between *POD* expression and time post inoculation, data were fitted with a second-degree polynomial regression model under different CDNPs treatments (Figure 6B–E). In AMV-inoculated control plants, polynomial regression between *POD* expression and time post inoculation was very weak (R^2^ = 0.0163 and *p* = 0.8840; Figure 6B). However, the relationship between them followed a positive and quadratic model when *N. glutinosa* plants were treated with protective (R^2^ = 0.3500 and *p* = 0.0395; Figure 6C), curative (R^2^ = 0.5245 and *p* = 0.0038; Figure 6D), or inactivating treatment (R^2^ = 0.4658 and *p* value = 0.0091; Figure 6E). Previous studies showed that chitosan induced the POD activity in *Pinus koraiensis* seedlings to its highest peak at 2 dpi [82]. Nevertheless, POD activity reached its highest levels at 9 hpi in downy mildew-infected pearl millet plants after the treatment with chitosan nanoparticles [83]. It is worth mentioning that the AMV-infected plants showed no increase in the transcript levels of POD compared with the mock-inoculated control. However, POD was upregulated only when plants were treated with CDNPs. Collectively, these findings indicate the priming effect after the application of CDNPs.

The obtained results showed that the prepared CDNPs might reduce ROS adverse effects on plant cell membranes by activating ROS scavenging enzymes. Therefore, the antioxidant properties of the prepared CDNPs can putatively increase resistance to oxidative stress in plant tissues. These properties are mostly owing to their abundance of active hydroxyl and amino groups, which can interact with ROS to generate stable and relatively non-toxic macromolecular radicals [84]. The obtained results agree with Chandra et al. [19], who reported that the application of chitosan or chitosan nanoparticles can potentially provide protection to the plants against different oxidative stresses through activating antioxidant enzymes that result in ROS inhibition. Furthermore, Choudhary et al. [85] suggested that increased *SOD* and *POD* activities following nanoparticle application may be responsible for scavenging ROS to protect plants from oxidative stress during pathogen invasion.

##### Pathogenesis-Related Protein 1 (PR-1)

**Figure 7:**
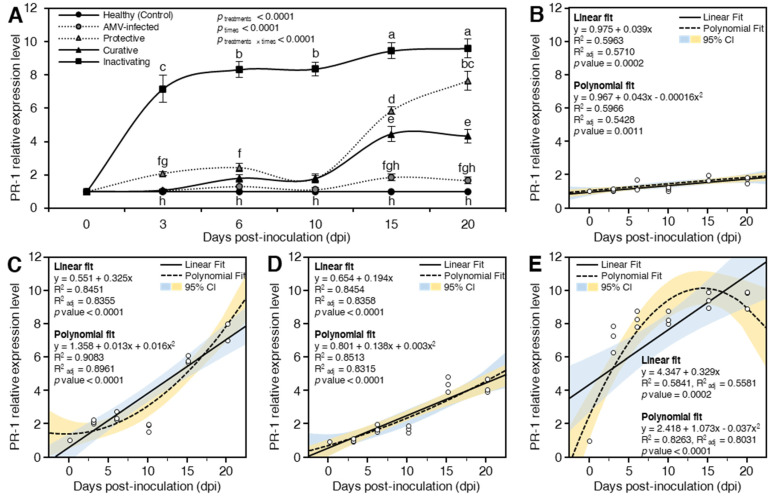
Effect of chitosan/dextran nanoparticles (CDNPs) on the relative expression level of Pathogenesis-related protein 1 (*PR-1*) in AMV-infected *N. glutinosa* plants at 3, 6, 10, 15, and 20 dpi. (**A**) *PR-1* relative expression levels of different treatments at 3, 6, 10, 15, and 20 dpi. Data presented are means ± standard deviation (mean ± SD) of three biological replicates. Different letters indicate statistically significant differences among treatments according to Tukey’s honestly significant difference test (*p*_time × treatment_ < 0.05). (**B**–**E**) Simple linear regression (SLR) and quadratic polynomial regression analysis between *PR-1* relative expression and time post-inoculation of AMV-infected, protective-treated, curative-treated, and inactivating-treated plants, respectively. Open small circles present the row data (*n* = 3), solid lines present the SLR line, while polynomial regression models are presented as dashed-line. The 95% confidence intervals for the estimated regression are blue- and yellow-shaded. Regression equations, R^2^, R^2^_adj_, and *p*-value based on the F test (*p* < 0.05) were also obtained and presented within the graph.

Salicylic acid (SA) is an important signal plant phytohormone molecule of SAR [86]. Furthermore, *PR-1* is a marker of the SA signaling pathway that inhibits programmed cell death, stimulates plant immunity, regulates SAR in plants, and may serve as a marker for early plant defense responses [87,88]. The accumulation and expression of *PR-1* is associated with the activation of SA in response to pathogens [89]. In the present study, both treatments (*p*_treatments_ < 0.0001) and time post-inoculation (*p*_times_ < 0.0001) significantly affected the relative expression levels of *PR-1*. The highest expression level of *PR-1* was reported in the inactivating treatment followed by protective treatment and curative treatment compared to mock-inoculated control and AMV-inoculated control plants at all tested time intervals (Figure 7A). At 3 dpi, the *PR-1* was significantly (*p*_treatments × times_ < 0.0001) up-regulated in protective and inactivating only with relative expression levels 2.09- and 7.16-fold higher, respectively, than the mock-inoculated control. The two treatments (AMV-inoculated control and curative) did not differ significantly (*p*_treatments × times_ < 0.0001) when compared to the mock-inoculated control (Figure 7A). These results suggest that the early induction of *PR-1*, which is associated with the accumulation of SA content, in protective and inactivating plants reflects the ability of CDNPs to develop plant SAR [19]. At 6 dpi, the expression levels of protective and inactivating continued to increase, while AMV-inoculated control and curative plants exhibited slight induction with relative transcriptional levels 1.31- and 1.80-fold greater than mock-inoculated control. Compared to the expression profile at 6 dpi, all transcriptional levels of all treatments decreased, apart from inactivating treatment, which showed an 8.36-fold increase in expression at 10 dpi (Figure 7A). This drop in the relative expression levels might be due to the increasing suppressor activity of AMV [31]. At 15 dpi, the highest expression levels (9.44-fold) were shown in inactivation followed by protection (5.84-fold), curative (4.45-fold), and AMV-inoculated control plants (1.83-fold) higher than mock-inoculated control (Figure 7A). At 20 dpi, the expression levels of protective and inactivating continued to increase, reaching their maximum levels, while AMV-inoculated control and curative plants exhibited a slight reduction in their relative transcriptional levels (Figure 7).

To better understand the relationship between relative expression of *PR-1* and time post-inoculation, data were fitted using an SLR model. Although the relative expression of *PR-1* was positively correlated with time post-inoculation in AMV-inoculated control plants, their relationship was weak (R^2^ = 0.5963 and *p* = 0.0002; Figure 7B). Nevertheless, the protective (R^2^ = 0.8451 and *p* < 0.0001; Figure 7C) and curative (R^2^ = 0.8454 and *p* < 0.0001; Figure 7D) significantly strengthened the correlation between relative expression of *PR-1* and time post-inoculation, even better than the inactivating treatment (R^2^ = 0.5841 and *p* = 0.0002; Figure 7E). In this study, relative expression of *PR-1* was positively correlated with time post-inoculation in AMV-infected plants. Nevertheless, CDNPs application significantly strengthened the correlation between relative expression of *PR-1* and time post-inoculation. It is worth mentioning that inactivating treatment had the highest PR1 levels over the time course. This might be due to the positive effect of CDNPs on the AMV particles, resulting in completely inactivated viral activity. Moreover, because of the nonlinear relationship between relative expression of *PR-1* and time post-inoculation, data were fitted using a second-degree polynomial regression model and presented in Figure 7B–E. It is worth mentioning that the relationship between relative expression of *PR-1* and time post-inoculation at different treatments followed a positive and quadratic model. The relationship between exogenous *PR-1* and time post-inoculation of AMV-inoculated control plants is described by the equation (R^2^ = 0.5966 and *p* = 0.0011; Figure 7B), while for protective-treated plants is described by the equation (R^2^ = 0.9083 and *p* < 0.0001; Figure 7C), for curative-treated plants (R^2^ = 0.8513 and *p* < 0.0001; Figure 7D), and for inactivating-treated plants is described by (R^2^ = 0.8263 and *p* < 0.0001; Figure 7E).

The transcriptional obtained results suggest that the application of CDNPs can be used as an eco-friendly approach to trigger plant immune defense systems that may result in SA content accumulation and SAR activation. It is well proven that PR genes are key components of the plant’s innate immune system, particularly SAR, and are commonly employed as diagnostic molecular markers for defense signaling pathways [90]. Thus, the overexpression of *PR-1*, either separately or in combination, can significantly increase plant defense against AMV infection. Previous studies have been demonstrated that overexpression of *PR-1* protein in tobacco plants increased resistance against TMV [91]. Moreover, the application of chitosan nanoparticles (0.1, *w/v*) was associated with the induction of several defense-related enzymes, including chitinase, protease inhibitors, *POD*, and *PPO* in finger millet plant leaves, which ended with boosting innate immunity [23].

##### Phenylalanine Ammonia-Lyase (PAL)

**Figure 8:**
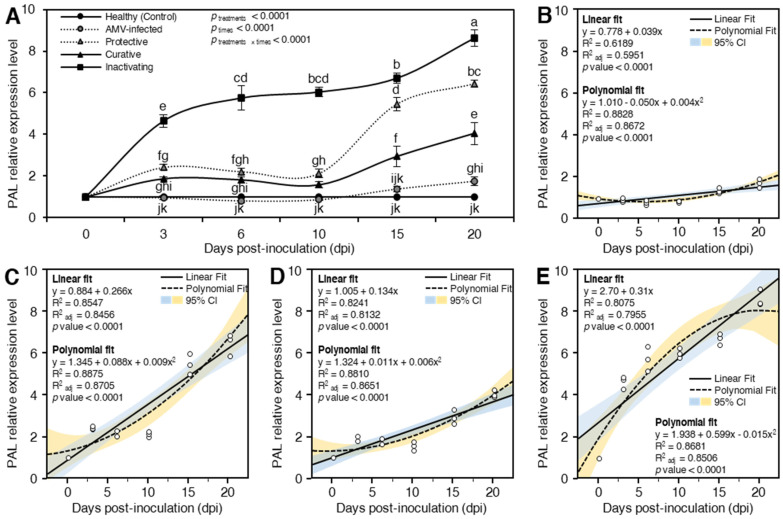
Effect of chitosan/dextran nanoparticles (CDNPs) on the relative expression level of phenylalanine ammonia-lyase (*PAL*) in AMV-infected *N. glutinosa* plants at 3, 6, 10, 15, and 20 dpi. (**A**) *PAL* relative expression levels of different treatments at 3, 6, 10, 15, and 20 dpi. Data presented are means ± standard deviation (mean ± SD) of three biological replicates. Different letters indicate statistically significant differences among treatments according to Tukey’s honestly significant difference test (*p*_time × treatment_ < 0.05). (**B**–**E**) Simple linear regression (SLR) and quadratic polynomial regression analysis between *PAL* relative expression and time post-inoculation of AMV-infected, protective-treated, curative-treated, and inactivating-treated plants, respectively. Open small circles present the row data (*n* = 3), solid lines present the SLR line, while polynomial regression models are presented as dashed lines. The 95% confidence intervals for the estimated regression are blue- and yellow-shaded. Regression equations, R^2^, R^2^_adj_, and *p*-value based on the F test (*p* < 0.05) were also obtained and presented within the graph.

Besides its role in SA biosynthesis, *PAL* is the key enzyme of the phenylpropanoid pathway, which connects primary and secondary metabolism by converting L-phenylalanine to ammonia and trans-cinnamic acid [92,93]. In higher plants, SA is synthesized from chorismate using isochorismate synthase [94] or from L-phenylalanine by the activity of *PAL* [95,96], which is more common. Moreover, it was stressed that the importance of phenolic compounds in disease resistance, as well as their accumulation through the phenylpropanoid pathway as a result of different elicitor applications, has been previously reported [11,19]. Like the *PR-1* expression profile, the highest transcriptional levels of *PAL* were observed in inactivating (*p*_treatments_ < 0.0001) treatment, followed by protective and curative treatments at all dpi (Figure 8A). Likewise, time post-inoculation significantly affected the *PAL* expression levels in different treatments (*p*_time_ < 0.0001). For instance, at 3 dpi, it was observed that the foliar application of CDNPs rapidly induced *PAL* with significant (*p*_treatments × times_ < 0.0001) relative expression levels 1.88-, 2.41-, and 4.64-fold higher than the mock-inoculated control in curative, protective, and inactivating treatments, respectively (Figure 8A). On the other hand, no significant difference was observed in *PAL* expression levels in AMV-inoculated control plants compared to mock-inoculated control at the same time. At 6 and 10 dpi, inactivating treatment plants exhibited dramatically increased *PAL* transcripts, while the three treatments (AMV-inoculated control, protective, and curative) showed dramatically decreased *PAL* expression levels at the same two-time intervals (Figure 8A). The AMV-inoculated control plants showed significant down-regulation with an expression level 0.82- and 0.87-fold lower than mock-inoculated control at 6 and 10 dpi, respectively. After that, the accumulation of *PAL* increased for all treatments, reaching a maximum at 20 dpi (Figure 8A). At 20 dpi, the relative transcriptional levels were 1.75-, 4.03-, 6.43-, and 8.62-fold higher than mock-inoculated control in AMV-inoculated control, curative, protective, and inactivating treatments, respectively. In this regard, the antiviral activities of CDNPs have been ruled out in AMV-inoculated control *N. glutinosa* leaves, and the inhibitory effects of treatment (protective, curative, and inactivating) were attributed to the elicitation of the plant defense mechanisms and direct suppression of viral replication [23,51,56,58]. In agreement with these findings, previous studies showed that although the Cucumber mosaic virus did not significantly affect the expression levels of *PAL*, chitosan reduced the viral load and up-regulated *PAL* expression in infected tomato plants [51]. Nevertheless, the expression profile of *PAL* usually depends on the host, pathogen, and maybe some other factors. For instance, *PAL* reached its highest activity at 2 dpi in *Pinus koraiensis* seedlings treated with 100 mg L^–1^ chitosan [82], while it peaked at 6 hpi in downy mildew-infected pearl millet plants after the treatment with chitosan nanoparticles [83].

Additionally, the relationship between relative expression of *PAL* and time post inoculation was fitted using an SLR model. Although the SLR showed that *PAL* expression was weakly correlated with time post inoculation in AMV-inoculated control plants (R^2^ = 0.6189 and *p* < 0.0001; Figure 8B), CDNPs application significantly strengthened the positive correlation between *PAL* expression and time post inoculation in protective-treated (R^2^ = 0.8547 and *p* < 0.0001; Figure 8C), curative-treated (R^2^ = 0.8241 and *p* < 0.0001; Figure 8D), and inactivating-treated plants (R^2^ = 0.8075 and *p* < 0.0001; Figure 8E). Our findings showed that *PAL* expression levels were slightly increased over the time course in AMV-infected plants compared with the healthy control. However, *PAL* gradually upregulated upon CDNPs application throughout the experiment (up to 20 dpi). Moreover, *PAL* expression levels were in a tandem match with the expression patterns of *PR1* over the time course of 20 dpi. Furthermore, to better understand the nonlinear phenomena between relative expression of *PAL* and time post inoculation, data were fitted with a second-degree polynomial regression model. Interestingly, the relationship between *PAL* expression and time post inoculation followed a positive and quadratic model in all tested treatments including AMV-inoculated control (R^2^ = 0.8828 and *p* < 0.0001; Figure 8B), protective-treated (R^2^ = 0.8875 and *p* < 0.0001; Figure 8C), curative-treated (R^2^ = 0.8810 and *p* < 0.0001; Figure 8D), and inactivating-treated plants (R^2^ = 0.8681 and *p* < 0.0001; Figure 8E).

The function of phenolic compounds in disease resistance, as well as their accumulation by the phenylpropanoid pathway as a result of various elicitor treatments, has previously been described. In the present study, the *N. glutinosa* plants showed a higher accumulation of phenolic compounds than mock-inoculated control and AMV-inoculated control plants, which could be a direct result of the induction and upregulation of *PAL* activity in the CDNPs treated plants. Consequently, CDNPs may work as an elicitor molecule that induces and stimulates the plant immune defense system, possibly inducing the early SA signaling pathway resulting in SAR activation [97]. Such a result is in accordance with Mejía-Teniente et al. [74], who observed that chitosan-treated *Capsicum annuum* plants exhibited increasing *PAL* expression. Moreover, Chandra et al. [19] reported that the treatment of tea leaves with chitosan nanoparticles was associated with an up-regulation of *PAL* activity that resulted in a higher accumulation of phenolic compounds. These data support the accumulation of more flavonoids in the chitosan-treated leaves, as well as a secondary defensive induction in the treated plants [19]. The intrinsic positive charge of chitosan-based nanoparticles can interact with the negative charge found on the cell membrane and mucosal surfaces, resulting in mucoadhesive characteristics. Moreover, the antiviral activity of nanoparticles may be due to the inactivation of viral multiplication and the activation of plant defense systems that lead to plant immunity [55].

In general, regression-based statistical modeling has been introduced previously in the biomedical field to identify the relationship between differentially expressed genes in time-course studies [98]. Regression-based prediction of gene expression patterns could be achieved using linear regression [99,100] and/or logistic regression [101] models. Moreover, polynomial regression methods, particularly quadratic regression analysis, are helpful in the identification of differentially expressed genes in a non-cyclic short-time course [102]. In the current study, we used simple linear (fitting a straight line to the observed data) and quadratic-polynomial (fitting a curved line to the observed data) regression modeling to better understand the quantitative relationship between gene expression and short-time course. Regression models of *POD*, *PR-1*, and *PAL* showed that all CDNPs treatments strengthened the positive relationship between gene expression and time-post inoculation as expressed by higher coefficients of determination (R2). In the activating treatment (premixing of viral particles with CDNPs for 1 h before inoculation), the absence of viral symptoms on the inoculated plants, negative ELISA results, and the highest induction of the three tested genes at all study times suggested that the inactivating treatment completely disrupted viral particles and inhibited replication. As a result, the inactivating treatment plants exhibited the highest transcriptional levels. On the other hand, the regression models suggested that the both protective and curative treatments showed a stronger correlation with time-post inoculation for POD, *PR-1,* and *PAL*. Collectively, our findings suggest that the protective and curative treatments, particularly protective, might be good for long-term solutions. However, more research into its ability under different combinations of host–pathogen interaction, as well as long-term (more than 20 days) studies, is needed to investigate the potential mechanisms and biological phenomena of differentially expressed genes over time.

## 4. Conclusions

In the current study, the antiviral activities of synthesized chitosan/dextran nanoparticles (CDNPs) on *N. glutinosa* plants were evaluated. Scanning electron microscope analysis revealed that the synthesized CDNPs were uniform, regular sphere shapes. In addition, particle size analysis reported that CDNPs were distributed in a range of 20–160 nm with an average diameter of 91.68 nm. Under greenhouse conditions, the foliar application of CDNPs (100 µg mL^−1^) decreased viral disease severity, induced systemic acquired resistance (SAR), reduced AMV accumulation levels, and up-regulated the transcriptional levels of *POD*, *PR-1*, and *PAL* genes. In light of the information gathered, we propose that the potential application of CDNPs could be a long-term and financially viable strategy for obtaining nutritional security.

## Figures and Tables

**Table 1:** List of the nucleotide sequences of the qRT-PCR primers used in this study.

Primer Name	Abbreviation	Direction	Sequence (5′…………………3′)
Alfalfa mosaic virus-coat protein	*AMV-CP*	Forward	CCATCATGAGTTCTTCACAAAAG
		Reverse	TCGTCACGTCATCAGTGAGAC
Peroxidase	*POD*	Forward	TGGAGGTCCAACATGGCAAGTTCT
		Reverse	TGCCACATCTTGCCCTTCCAAATG
Pathogenesis related protein-1	*PR-1*	Forward	GTTCCTCCTTGCCACCTTC
		Reverse	TATGCACCCCCAGCATAGTT
Phenylalanine ammonia-lyase	*PAL*	Forward	GTTATGCTCTTAGAACGTCGCCC
		Reverse	CCGTGTAATGCCTTGTTTCTTGA
Beta-actin	*β-actin*	Forward	TGGCATACAAAGACAGGACAGCCT
		Reverse	ACTCAATCCCAAGGCCAACAGAGA

## Data Availability

All data reported here are available from the authors upon request.
